# Informal teacher communities enhancing the professional development of medical teachers: a qualitative study

**DOI:** 10.1186/s12909-016-0632-2

**Published:** 2016-04-14

**Authors:** Thea van Lankveld, Judith Schoonenboom, Rashmi Kusurkar, Jos Beishuizen, Gerda Croiset, Monique Volman

**Affiliations:** Faculty of Behavioural and Movement Sciences, LEARN!, VU University Amsterdam, Amsterdam, Netherlands; VUmc School of Medical Sciences Amsterdam, LEARN!, Amsterdam, Netherlands; Faculty of Social and Behavioural Sciences, Research Institute of Child Development and Education, University of Amsterdam, Amsterdam, Netherlands

**Keywords:** Teacher community, Faculty development, Professional development, Teachers, Peer learning, Identity

## Abstract

**Background:**

Informal peer learning is a particularly powerful form of learning for medical teachers, although it does not always occur automatically in the departments of medical schools. In this article, the authors explore the role of teacher communities in enhancing informal peer learning among undergraduate medical teachers. Teacher communities are groups of teachers who voluntarily gather on a regular basis to develop and share knowledge. Outside of medical education, these informal teacher communities have proved to be an effective means of enhancing peer learning of academic teachers. The processes underlying this outcome are, however, not known. This study therefore aims to explore the processes that make informal teacher communities effective in supporting peer learning of teachers.

**Methods:**

A qualitative study was performed at a Dutch medical school, where a student-centred undergraduate curriculum had recently been introduced. As part of this curriculum, tutors are segregated into separate specialty areas and thus have only limited opportunities for informal learning with other tutors. The authors followed two informal teacher communities aimed at supporting these tutors. They observed the interactions within the teacher communities and held semi-structured interviews with ten of the participants. The observation notes and interview data were analysed using thematic analysis.

**Results:**

The informal teacher communities allowed the tutors to engage in a dialogue with colleagues and share questions, solutions, and interpretations. The teacher communities also provided opportunities to explicate tacit expertise, which helped the tutors to develop an idea of their role and form a frame of reference for their own experiences. Furthermore, the communities enhanced the tutors’ sense of belonging. The tutors felt more secure in their role and they felt valued by the organisation due to the teacher communities.

**Conclusions:**

This study shows that informal teacher communities not only support the professional development of tutors, but also validate and strengthen their identity as teachers. They seem to provide a dialogical space where informal intercollegiate learning is stimulated, stories are shared, tacit knowledge is made explicit, concerns are shared, and teacher identity is nurtured.

**Electronic supplementary material:**

The online version of this article (doi:10.1186/s12909-016-0632-2) contains supplementary material, which is available to authorized users.

## Background

Many medical teachers have never been trained to teach. This becomes especially problematic when they come to teach in student-centered curricula, which require specific skills, like facilitating active and self-directed learning of students. Medical teachers usually learn these facilitating skills informally from peers in the situated social practices of the everyday workplace. Since the informal learning that occurs from peers is crucial to the professional development of medical teachers, several authors have argued that more research needs to be conducted on peer learning [1–3]. One of the theories that has drawn particular attention to the socialisation processes of novices is situated learning theory [4, 5]. This theory argues that newcomers develop expertise through increasing participation in and interaction with a community of professionals. In the initial phase, newcomers participate on the professional periphery, where they complete relatively low-risk tasks. Meanwhile, they have legitimate access to the social practices of the community and so learn what it takes to be a professional, including the unspoken rules, expectations concerning competence, professional values, and so on. Informal learning from more experienced colleagues and from peers is a crucial component of this situated learning. Newcomers learn through talking with colleagues, observing their behaviour and hearing their stories.

Empirical research on the actual practice of the peer learning of medical teachers, however, paints a different picture than the one described by situated learning theory. In the practice of teaching medicine, learning from colleagues and peers seems to be rather limited; feedback from colleagues is “rare and often unhelpful” [6, 7]. Instead, medical teachers seem to learn to teach in an isolated way: from individual reflection on their on-the-job experiences of teaching and from reflection on earlier negative and positive experiences from their student life [6, 7]. In other disciplines within higher education, similar findings have been reported. Novice academics often find collegial support to be limited and they commonly feel they are left to find their own way into the practice of teaching [8, 9]. Opportunities for peripheral participation and starting with relatively easy tasks are limited too: most academics are immediately immersed in intense practice and hence experience a feeling of “being thrown into the deep end” [8, 10–12]. Opportunities to observe the modelling of good practices and collaboration are limited [10, 13, 14], while the discussion of teaching tends to focus on what is taught, rather than on how it is taught [8, 9]. Research has shown that interactions concerning teaching are concentrated within departments, and that informal learning beyond departmental boundaries is limited [15, 16]. Overall, the workplaces of medical teachers in particular and university teachers in general seem to provide only limited opportunities for informal learning.

Some have argued that the limited opportunities for peer learning are related to the tacit nature of teaching expertise. As teaching expertise is grounded in professional activity rather than in simple rules, it cannot easily be expressed [17]. The tacit nature of teaching expertise makes it hard for novices to capture, unless a dialogical space is created in which they can make this tacit expertise explicit. Such a dialogical space can be created in several ways, for example through collective decision making, reflecting in and on practice in reflective dialogue, or by engaging in collaborative inquiries into practice [18]. These dialogues allow for the sharing of ideas concerning successful and unsuccessful teaching approaches, as well as for explicating interpretations and underlying reasoning, which can lead to a rich mix of perspectives for teachers to draw upon. Dialogue can also enhance innovation, creativity and emotional support.

One way to create a dialogical space is to foster teacher communities, i.e. informal groups of teachers who gather voluntarily and regularly in order to develop and share knowledge with and from each other [19]. In his later work with McDermott and Snyder [20], Wenger argues that when communities of practice are weak and the potential of peer learning is not fully achieved, communities can be created or cultivated by paying attention to the three constituent characteristics of communities of practice:

- The *domain* that members share, with recurring sets of problems. The domain is the cohesive factor in a community, i.e. what brings the members together. The community’s sense of identity is rooted in this shared domain.
- The *community*, in the form of the interactions and relations between the members. The continuity and quality of interactions between members determines their willingness to share questions and ideas.
- The *practice* is the understanding of the domain that the community members share, including approaches, frameworks, ideas, standards, styles, language, stories, theories, models, principles, and so on. This ranges from tangible objects, like tools and documents, to less concrete displays of competence, such as the ability to recognise a certain problem.

Unfortunately, Wenger uses the phrase “community of practice” for both the natural communities (as described in his 1998 work) and the cultivated, developed communities he described in his later work with McDermott and Snyder in 2002. The use of the same label for two different contexts may lead to confusion. In order to avoid this confusion, we adopted the label *teacher communities* in this study.

Teacher communities may be particularly helpful in fostering the informal peer learning among tutors especially in the early phases of the implementation of undergraduate student-centered or problem-based curricula [21, 22]. New tutors have difficulties understanding the process of student-centred or problem-based learning, struggle with the kind of facilitating behaviours they are expected to display [23] and need opportunities to reflect on the dynamics of their student groups [22]. Since these tutors are often segregated into separate specialty areas [24], they have only limited opportunities for informal contacts with other tutors.

No research has yet been reported on teacher communities in the field of medical education. This is surprising, since several authors have ascribed to the potential of teacher communities [1, 22–24]. In other domains within higher education, informal teacher communities have been shown to lead to a diversity of outcomes for teachers [25, 26]. First, they support teachers’ learning and help them to improve their teaching practice. Moreover, teacher communities remove barriers to change and generate new and innovative teaching strategies [25, 26]. Second, teacher communities have been shown to lead to enhanced collegiality and a sense of belonging [26]. Third, teacher communities have been found to empower those with an intrinsic motivation for teaching as well as to provide a morale boost for disillusioned staff [25].

The existing studies on teacher communities do not give insight into the processes that make informal teacher communities effective. Regehr [27] recommends that medical education research should not only focus on the effects of interventions, but also explore the processes behind these effects. Focusing on the processes in this case would provide better insight into the working of teacher communities.

This study investigated the role of informal teacher communities in enhancing the informal peer learning among tutors in an undergraduate student-centered curriculum that was in its early stage of implementation. The research question guiding our study was: “What are the processes that make informal teacher communities effective in supporting the professional development of teachers?”

## Methods

### Design

In order to study the processes involved in teacher communities, we employed a qualitative approach. The processes were observed. In order to understand how the participants had experienced the processes, semi-structured interviews were conducted with the tutors.

### Setting

The study took place at a Dutch medical school that offers a 3-year bachelor’s programme and a 3-year master’s programme [28]. Two years before the study was conducted, the school had introduced a new vertically integrated student-centred bachelor’s curriculum. Small group learning was introduced into the first and second years of the course, with students meeting in groups of 12 for two sessions a week. In the first session, the students brainstorm about assigned questions related to cases, while in the second session, they present the answers to these questions to each other, based on their self-study and mutual collaboration during the previous days. Tutors facilitate the learning process of the small groups and assess the professional behaviour and presentation skills of the students.

Clinical departments as well as non-clinical departments (e.g. biomedical, social sciences, public health), were required to provide a certain number of tutors to guide small groups (depending on the size of the department), for which these departments received remuneration. Departments differed in the way they selected tutors; at some departments tutors volunteered, at others they were assigned to the task. Before starting their task, the new tutors attended a 2-day preparatory training course to acquire the necessary skills. In the first day of the training, the student-centered curriculum and the role of the tutor were explained. Through discussions and role plays, the tutors practiced and reflected on the skills required for facilitating self-directed learning in small groups.

The role of “tutor” was new to the medical school, although some departments had previously employed small group learning. Not all staff members were enthusiastic about the change of the role of the teacher from expert to facilitating tutor. During the first 2 years of the new curriculum, many tutors struggled with their new role. Many were confused about the type and amount of guidance they should give to the students, and struggled with how to assess students’ professional behaviour. Some also struggled with the perceived low status of the tutor role among their colleagues.

### The teacher communities

Since almost all tutors came from different departments and access to other tutors therefore was not easy, one of the teachers took the initiative to organise teacher communities in which tutors could support and learn from each other. The organising teacher asked the first author (TvL), a professional educator, to be involved. The medical school approved the initiative and supported the teacher communities by providing lunch for all participants and a room for them to meet in (a scarce resource at this medical school). Based on voluntary participation, two communities were formed: one for tutors in year 1 of the bachelor programme and one for the tutors in year 2.

The teacher communities were organised according to Wenger, McDermott and Snyder’s approach [20], which means that attention was paid to the three constituent characteristics of communities of practice: having a shared domain, being a community and understanding the practice.

- The *shared domain* consisted of the shared experience of being a tutor in a recently introduced student-centred curriculum. In the teacher communities, the tutors reflected on shared problems they had experienced and exchanged possible solutions. Each meeting was led by an alternating pair of tutors, who chose a topic and decided how to discuss it. Thus, the topics were directly related to the tutors’ working practice and held a sense of urgency for them. The topics that were chosen concerned the pedagogical part of the tutor role. (See Table 1 for a description of the meetings of the two groups).
- *Community*: Lively interaction was stimulated by organising 5 monthly lunchtime meetings. Discussions were based on case stories, thematic questions, video recordings of their student groups, or role plays. Examples of questions that were discussed are: “How to facilitate the brainstorming process?” and “How to assess the students’ professional behaviour?”
- *Practice*: The tutors’ understanding of how to enact the tutor role was developed by stimulating their collaborative learning. Reflective dialogue was stimulated by encouraging the participants to reflect in depth on the possible backgrounds to the questions and problems that were put forward, as well as by stimulating them to collaboratively explore a rich diversity of possible solutions. After a year, the outcomes of the communities were collected into a tangible document entitled “Tips for tutors”, which was shared with all the tutors at the medical school.

**Table 1:** Overview of the meetings

Group 1 and Group 2	
1. *Start-up* The aim of the start-up meeting of both groups (prepared by the facilitators) were to get acquainted with each other and the goal of the teacher community. The initiative received acclaim from the tutors. Possible questions to discuss this semester were explored. Most mentioned questions were: How to give shape to the tutor role? How to assess students’ professional behaviour? The tutors were about to meet their student groups for the first time. They shared suggestions on how to start this first meeting.	
Group 1	Group 2
2. *Structured discussion of two videos* This meeting was prepared by two tutors, who had both made a video-recording of their student group, in which the students were expected to brainstorm (activate prior knowledge). Each of the tutors showed ten minutes of the video, and provided questions for the other tutors to observe it. The brainstorm in one video appeared to be rather chaotic, the other rather passive. The question that was discussed was what a tutor can do to stimulate the student-chair to take his/her role. Six suggestions were shared.	2. *Structured reflection on an actual situation* The two preparing tutors brought in an actual case with which all tutors had wrestled that week: how to deal with resistance of students as a result of a change of policy by the organization? It turned out that the tutors themselves experienced resistance to this change as well. A structured reflection method was used to analyze the case. Six suggestions were shared how to deal with situations like these and agreements were made to take collective action.
3. *Case discussion (session)* The preparing tutor brought in a case about a session in which students gave presentations, about which the tutor was not satisfied. The tutors collaboratively analysed the two underlying reasons and provided multiple suggestions for both.	3. *Role play* The two preparing tutors chose the theme, “how to facilitate the weekly brainstorm sessions”, for this meeting. First, the tutors shared successes and difficulties on the white board. The group then did a short role play of a stagnant brainstorm. After the role play, possible causes were discussed, and multiple suggestions were exchanged. The tutors agreed to try some of these suggestions in their own groups coming weeks.
4. *Successes and feedback to the organization* As the preparing tutors were late, the facilitators invited the other tutors to concisely share their successes of last month. Also two concerns were shared that proved to be relevant to all tutors. Actions were formulated about reporting this feedback to the organization. *Case discussion (student)* One of the two preparing tutors presented a case about the difficult behavior of one student. Several approaches were discussed for how to deal with this in the assessment of professional behavior that was planned for that week.	4. *Looking back* First, the tutors reflected on suggestions given in last meeting which they had tried in their own groups. Three positive experiences were reported. *Structured discussion of a video and a text* The two preparing tutors had chosen *\*as the theme of this meeting. They showed a short video of a secondary school group. The tutors then analysed underlying problems and provided suggestions for possible solutions. Duos of tutors then read one part of a text on group dynamics. Each duo summarized the paragraph for the rest of the group. Finally, the group discussed the relevance of the text to their own situation.
5. *Start-up/evaluation* This meeting was the last meeting of this group, and at the same time the first meeting of next semesters’ group, so both experienced and new tutors were present. The aim of the teacher community was clarified, and experienced tutors shared what the teacher community had brought them last semester. *Reflection on the role of tutor* The preparing tutor reflected on the processes in his last semester’s group, with which he was not completely satisfied. He wondered what he could have done better. The tutors explored the case and together formulated several suggestions, which also helped the new tutors to prepare for their role.	5. *Start-up/evaluation* Like in group 1, both experienced and new tutors were present, as this was a combined start-up and evaluation meeting. The aim of the teacher community was clarified, and the experienced tutors shared what the teacher community had brought them last semester. *How to start?* The new tutors, who were about to meet their student groups for the first time, felt the urgent need to hear from the experienced ones how to start this first meeting. Several suggestions were shared. *Reflection on role of tutor* The two preparing tutors had chosen the theme “*what went well last semester?*” for this meeting. They asked the group to first reflect on the question, “what is a good tutor?”, and then invited them to share the interventions that had proven to be successful in that semester. Nine successful interventions were shared.

The teacher who initiated the groups facilitated the teacher communities together with the first author. They sent invitations, organised the location, and ensured that a pair of tutors was chosen to prepare the next meeting. One important task was to monitor the cohesion of the group, so as to ensure that the tutors felt safe to raise questions or problems during the meetings. The facilitators participated in the discussions, and they sometimes provided relevant educational literature.

### Participants

About 30 tutors from both clinical and non-clinical departments were invited to participate in the communities by e-mail. Nineteen accepted the invitation: ten tutors from year 1 and nine tutors from year 2. Most of these tutors did not know each other prior to the teacher community. All participants in the teacher communities were invited to voluntarily participate in the interviews. Ten agreed (four tutors from the first year of the bachelor’s programme and six tutors from the second year), while nine declined because of time constraints. Demographic details regarding the participants are presented in Table 2.

**Table 2:** Demographic details of the participants

	Participants (teacher communities)	Participants (interviews)
Participating tutors		
Year 1	10	4
Year 2	9	6
Gender		
Male	6	2
Female	13	8
Experience as a tutor		
1 or 1.5 year(s)	6	5
Novice tutor	13	5
Teaching experience		
Yes	12	6
No	7	4
Background		
Medicine	10	6
Non-medicine	9	4
Department		
Clinical	9	5
Non-clinical	10	5

### Data collection

During the first semester, TvL took detailed observation notes during and immediately after the meetings of the two communities. As the processes we were looking for were unknown beforehand, we did not use a standardised observation scheme. Instead, we tried to include anything that seemed relevant to the research question from the perspective of the theoretical framework. The notes were qualitative in nature. They included: the content of the discussion, i.e. the questions brought in (domain) and the solutions offered (practice) and any side discussions, and the interactions between the members and any important events or incidents (community).

After the semester, TvL held individual semi-structured interviews with the participants.

TvL is a female educationalist holding an MSc in Psychology and extensive experience in qualitative methods. The participants were aware of the aim of the interview, namely to reflect on the personal outcomes of the teacher communities (both positive and negative) and on the processes within the teacher communities that had contributed to these outcomes. The interview schedule was developed by TvL and MV and is available online as Additional file 1. Questions were asked about being a tutor (domain), as well as in what ways the teacher community had contributed to their understanding of the tutor role (practice) and to contacts with other tutors (community). We also added two questions in which the participants were encouraged to explicate any negative experiences or things they had missed in the teacher community. The interview schedule was adapted after the first interview (the data of which has not been included in the analysis).

The interviews lasted for 60–90 min and took place at the university, without anyone else being present apart from the interviewer and the interviewee. Field notes were made during the interview. The interviews themselves were audiotaped and transcribed verbatim. Summaries of the interviews were sent to the participants for member checking.

### Analysis

Data analysis was performed from an interpretivist point of view [29], which fits well with our aim to construct insightful accounts of the processes involved in the teacher communities. First, TvL and JS analysed the observation notes. Through repeated reading of the observation notes and extended discussions concerning our interpretations, we identified two main processes in the teacher communities, which were derived from the data. In order to gain further understanding of the way the participants had experienced those processes, we analysed the interview data using thematic analysis [30]. We started with the two initial processes that we had identified in the observation notes as initial themes, but allowed for additional themes to emerge. The data was first systematically and iteratively analysed in Atlas.ti by TvL, and then re-analysed by JS. Any discrepancies were discussed until consensus was reached, which led to the further refinement of the analysis. In order to adequately interpret the interview data, we repeatedly went back over the original observation notes, so as to relate the participants’ accounts of the processes and their effects on them to the processes we had observed. The product of the analysis, a detailed description of three processes including illustrative data extracts and relevant contextual information, was discussed with the whole research team (i.e. triangulation of investigators [31]).

### Reflection on the role of the researchers

TvL played a double role due to both serving as a co-facilitator in the teacher communities and being involved in the data collection and analysis. She was therefore not a neutral observer, but was involved in the teacher communities herself. We acknowledge that this might have influenced the findings and our account of them. At the same time, we experienced that this double role actually helped us to understand the participants’ responses during the interviews, and it also helped us to recognise and understand the processes involved in the teacher communities during the analysis. To minimise possible conflicts between the two roles, a second researcher was involved in the data analysis, which assisted TvL in creating distance from her role as co-facilitator. Additionally, the research team, consisting of two scientists from the medical education field and three educational scientists, functioned as a critical review board during the data analysis.

## Results

In line with earlier studies, we found that the informal teacher communities supported the tutors’ learning and helped them to change their practice. This led to enhanced collegiality and a sense of empowerment. We identified three processes within the teacher communities that were involved in these outcomes: developing a frame of reference for the practice of tutoring; creating a sense of belonging; and valuing the domain of tutoring. Each of these processes is described in more detail below.

### Developing a frame of reference for the practice of tutoring

At the start of the semester, many of the tutors felt insecure about their role. The teacher communities helped them to gradually develop an understanding of what the tutor role entailed. Along the way, they also developed a frame of reference to which they could relate and gauge their own tutoring style and performance.

In the discussions concerning problems and difficult situations, a range of possible solutions was often exchanged. In general, the exchanged solutions were of a good quality, and if not, the tutors corrected each other by adding other solutions or other insights. In that way, the tutors collectively negotiated what counted as “good” or “valid” behaviour for tutors and what did not. The contributions of the experienced tutors were particularly helpful, since they could point out what solutions they had tried in the past, as well as what solutions had worked and what solutions had not. This helped the tutors to expand their repertoire.

The range of solutions offered also illustrated that there are many different ways in which a tutor can influence the learning process of the student group. This helped the participants to develop an understanding of the different ways of enacting the tutor role, while leaving it to individual tutors to choose the way that best fitted their own situation or preference. When exploring the underlying reasons for choosing one solution or another, the discussions often led to a consideration of the role of the tutor in enhancing students’ learning. These collaborative investigations of what the tutor role entailed helped the participants to make sense of the new concept of student-centred learning and their role in it. This increased their confidence:

*I do believe that I can now give shape to the tutor role, that I can play with the group and I can make sure that the group runs well. (Jacqueline, experienced tutor, clinical department).*

In the interviews, the tutors often mentioned that they found it helpful to know each other’s questions and interpretations of situations. When the interpretations and solutions were different from their own, the tutors re-evaluated their “usual” interpretation of the situation and they felt stimulated to try out different ones. This fuelled their creativity in finding solutions and so expanded their tutoring repertoire. When the problems or solutions were similar to their own, the tutors experienced a sense of recognition and emotional support. In the communities, the tutors realised that other tutors were grappling with similar or more difficult problems and that “it wasn’t just me.” The community thus constituted a source of confirmation of their doubts and helped them regain their sense of competence:

*You can mirror yourself, you can compare yourself with others. You realise that what others say, you thought about that yourself. You get a sort of confirmation of your qualities. (Tamara, novice tutor, clinical department).*

### Creating a sense of belonging

Most of the tutors were the only one in their department performing the role of tutor and so they felt rather isolated, having no one in their proximity to discuss their questions with. The teacher communities pulled them out of this isolation and provided them with a sense of belonging or a sense of community:

*You don’t feel alone. You have the idea that you’re not alone, but that you are part of a whole. (Jacqueline, experienced tutor, clinical department).*

What particularly contributed to this sense of belonging was the recognition that other tutors were experiencing similar problems and therefore understood the struggles that the participants saw themselves facing. In the meetings, the tutors also shared their concerns about the way the student-centred concept was implemented by the educational organisation and communicated to them and the students. They all felt that they were not being heard by the organisation, and that they were not being recognised for their expertise as tutors. Finding out that they all experienced these concerns and frustrations created a sense of belonging, and it helped the tutors to look for ways to take individual and collective action.

### Valuing the domain of tutoring

In the interviews, the tutors also mentioned that they viewed the establishment of the teacher communities as a signal that tutorship was taken seriously and considered an important task by the organisation:

*For me, the communities are a sign that the role of tutor is being taken seriously, that you are doing something that is important. (Florence, experienced tutor, non-clinical department).*

Thereby, the teacher communities counterbalanced the opinions of those clinician and scientist colleagues, who, according to the tutors, regarded tutorship as a “weak” job that “does not take much” because it did not seem to require content expertise. In and through the teacher communities, the tutors felt appreciated and valued in their role:

*People look down on tutorship, at least that’s the idea I have. Not every tutor is enthusiastic about it or takes it very seriously. But now that the teacher communities exist, it looks like the medical school does take it very seriously. (Danique, novice tutor, non-clinical department).*

Some of the tutors were disappointed that not *all* tutors came to the teacher communities; they interpreted their absence as a sign that these tutors did not take their responsibility as seriously as they did themselves. They sometimes found this rather demotivating.

## Discussion

Earlier research has shown that when attention is paid to the three fundamental elements of communities of practice (domain, community and practice), informal teacher communities can enhance the professional development of tutors in an undergraduate student-centred curriculum as well as lead to increased collegiality and a sense of empowerment [25, 26]. This study has shed light on the actual processes that generate these outcomes, which has previously been an under-researched field within medical education [2, 3]. We found three processes to be involved: developing a frame of reference for the practice of tutoring; creating a sense of belonging; and valuing the domain of tutoring. We will discuss each of these processes below in relation to the prior literature.

The first underlying process is that the informal teacher communities helped the tutors to build a frame of reference for the practice of tutoring. The teacher communities thus helped them to make sense of their role in the student-centred curriculum and in their acceptance of it [32]. Of particular help in this respect, was the sharing of stories concerning difficult situations and possible reactions to those situations. These stories contained the kind of tacit expertise that teachers use in their practice. We agree with other authors that the learning of teachers is based on *storytelling*, which can be described as the vicarious sharing of personal stories in which teachers describe professionally significant experiences [23, 33]. Stories are not only a means of understanding the complex practice of teaching [23], but also provide mirrors with which to validate one’s teaching practice [33]. In the teacher communities in our study, the stories functioned as resources to compare one’s own functioning to. In that way, the teacher communities provided the tutors with *stories to relate to*.

The second underlying process we found is that a sense of belonging was created in the informal teacher communities. Several aspects were involved in this. First, the teacher communities allowed the tutors to create connections with tutors from other departments. As in most student-centred or problem-based learning curricula [24], the tutors in this study were segregated into separate departments, and thus had only limited opportunities for informal learning. The teacher communities formed a platform where tutors who shared similar challenges and questions were brought together. Sharing their questions gave the tutors emotional support, since they recognised that they were not the only ones struggling. Second, the teacher communities provided a forum for the tutors to position themselves within local discussions taking place in the institution and to protect their shared interest. In the teacher communities, the tutors experienced how a problem they were faced with was also shared by others, for example in session two of group two (Table 1), in which the tutors realized they were all wrestling with the resistance of students as a result of a change of policy. This then helped them to identify individual and collective actions to solve it.

Finally, we found that the tutor role was valued and considered important in teacher communities. Valuing the role is important for the intrinsic motivation for teaching [25, 34]. In the teacher communities, the implicit and explicit messages about the tutor role were positive. This was opposed to the “hidden curriculum” of the culture of the workplace where, according to the teachers in the study, the tutor role was considered a “weak” job. The teacher communities thus validated and strengthened the tutors’ identity as a teacher. While the teacher identity was under pressure in departments where the research identity and/or clinical identity are dominant [35], in the teacher communities the teacher identity was appreciated. Hafler and colleagues [36] argued that faculty development programmes may function as a source of conflict when they are not in line with the “hidden curriculum” of the institutional culture. However, in our study we found that the teacher communities did not function as a source of conflict, but quite to the contrary, helped to resolve this conflict. They provided an alternative community for teachers where they felt appreciated and experienced a sense of connectedness with fellow teachers. It is interesting to note that the tutors felt valued by the organisation because of the teacher communities, when the initiative came from one of the tutors. Is seems valuable for an organisation to reward initiatives of teachers that meet their needs. As the study shows, supporting teachers’ initiatives might be as simple and inexpensive as providing a room and lunch.

### Limitations and suggestions for future research

Our study is limited to the role of informal teacher communities in enhancing the professional development of tutors in an undergraduate student-centred curriculum. As we have not investigated teacher communities for other teaching roles, our study has limited generalisability. However, our aim was to explore the processes that make teacher communities effective. We believe an exploratory study of two groups forms a good starting point for achieving this objective, although further research is needed to extend our work to other groups and contexts. Comparison of successful and less successful communities would be a valuable way to fully understand the processes that make teacher communities effective. It would also be worthwhile to explore the value of teacher communities for other roles than tutors in undergraduate student-centred learning (i.e. assessors, study material developers) or for clinical teacher roles in postgraduate settings. Positive experiences have been reported in the Dutch context [19, 37], but more research is needed to find out whether peer learning initiatives like teacher communities prove sustainable for clinical teachers who are confronted with several competing tasks, including teaching, research and patient care.

It is also relevant to point out that though the tutors themselves felt more secure in their role, we do not know whether the tutors actually performed better than those who did not participate in the teacher communities. This would also be an interesting question for further research. A further important point to note is that the two teacher communities in our study were active in a particular context, namely in the early stage (i.e. the third year) of a new curriculum. In this particular context, the organisation had not yet fully developed the tutor role at the time of the study.

The teacher communities are still active in our medical school, and are now facilitated by some of the active participants of the first groups. The number of participants is smaller than in the first years though. The teacher communities seem to be particularly appealing to novice tutors, who find a sense of belonging there and an opportunity for reflection, and less so for more experienced tutors, who set their priorities differently. Further research is thus needed to identify how long teacher communities can create value for the participants and for the organisation, as well as to determine what type of value is being created [38]. It would be interesting to follow teacher communities for a longer period of time than we did, in order to find out what is needed to keep teacher communities effective in the long run. We have strong cues that the teacher communities provided a fostering environment for tutors motivated to adopt coordination roles. It would be interesting to do further research on whether tutors who participated in the teacher communities were more likely to continue as tutors in subsequent years.

Depending on one’s point of view, the double role of researcher and co-facilitator could be viewed as either a limitation or a strength. We do acknowledge that the double role might have influenced the findings to some extent, but we also view it as a strength because it helped us to recognise and understand the processes involved in the teacher communities. We also believe that it helped us to ask supplementary questions during the interviews. We took measures to minimise the risk that participants would feel reluctant to mention any negative aspects of the teacher communities and to minimise the risk of biased interpretation during the analysis.

## Conclusions

This study shows that informal teacher communities not only support the professional development of tutors in student-centred curricula, but also validate and strengthen their identity as a teacher. They seem to provide a dialogical space where informal intercollegiate learning is stimulated, stories are shared, tacit knowledge is made explicit, concerns are shared, and the teacher identity is nurtured. A community around the shared practice of being a tutor is hard to find in the medical schools, but we found that it can be deliberately cultivated in the form of teacher communities. Our study showed how a theoretical framework like that of Wenger and colleagues [20] can inform teacher communities. When attention is paid to the three constituent characteristics of communities of practice [20], that is having a shared domain, forming a community and understanding the practice, teaching communities can be appropriate ways to support peer learning among medical teachers.

### Ethics approval and consent to participate

At the time of the study, approval from an ethical committee was not required for educational research in the Netherlands, and a national ethical review board for medical education research did not yet exist [39]. Participation in the study involved no risks to the participants. The participants of the teacher communities were informed about the study and agreed that the reports of the meetings would be used for research. All reports were approved by all participants. The participants who agreed to participate in the interview were informed of the aims, methods and confidentiality of the study prior to participation, and all gave informed consent. Participants were allowed to withdraw from the study at any time. In order to protect the privacy and confidentiality of the participants, no names were used in the observation reports and pseudonyms were used in the interview transcripts.

### Availability of data and materials

The interview schedule is available online as an additional file. Data will not be shared since we have not taken permission for this from the participants.

## Additional file

Additional file 1: Interview schedule. (DOCX 18 kb)
