# Supplementary material for: Informal teacher communities enhancing the professional development of medical teachers: a qualitative study
Source: BMC Med Educ. 2016 Apr 14;16:109. doi: 10.1186/s12909-016-0632-2 (PMC4832453; doi:10.1186/s12909-016-0632-2)
Supplement: Additional file 1: — Interview schedule. (DOCX 18 kb) [file 12909_2016_632_MOESM1_ESM.docx]

**Interview schedule**

*Clarification of the context*

*Earlier experiences as tutor and teacher*

1. Did you have experience as a tutor before you participated in the teacher community? If so, how many semesters have you been a tutor?
2. Did you have teaching experiences before you participated in the teacher community?

*Experiences of being a tutor*

1. In the beginning, what was it like for you, to be a tutor? What challenges were you confronted with? How did they affect you?
2. What is like for you to be a tutor now?
3. Do you see yourself differently, since the teacher community? In what respect?
4. Does being a tutor mean something different for you than other educational tasks you have? In what respect?

*Contribution of the teacher community to professional development*

*Contribution to understanding the domain*

1. What did the teacher community bring you? What did you learn? What (processes) in the teacher communities contributed to these outcomes?
2. What is the *most important* surplus value of the teacher community to you? What (processes) in the teacher communities contributed to these outcomes?
3. Which of the suggestions or insights you got from the teacher community were most helpful? Why?

*Contribution to interactions and relations between the community members*

1. Have you come to know other tutors because of the teacher community? Is that important to you? If so, in what respect?
2. Has your motivation for being a tutor changed during the teacher community? In what respect? What (processes) in the teacher communities contributed to these outcomes?

*Evaluation of the teacher community*

1. Would you recommend the teacher community to other tutors? Why (not)?
2. Have you missed anything in the teacher community? Could it have yielded more, you think? What?
3. Did the teacher community lead to any other negative experiences? Which? What contributed to these negative experiences?
